# α2β1 integrin affects metastatic potential of ovarian carcinoma spheroids by supporting disaggregation and proteolysis

**DOI:** 10.1186/1477-3163-6-11

**Published:** 2007-06-14

**Authors:** Kristy Shield, Clyde Riley, Michael A Quinn, Gregory E Rice, Margaret L Ackland, Nuzhat Ahmed

**Affiliations:** 1 Gynaecological Cancer Research Centre, Royal Women's Hospital, Melbourne Australia; 2 Centre for Cellular and Molecular Biology, Deakin University, Melbourne, Australia; 3 Translational Proteomics, Baker Heart Research Institute, Melbourne, Australia; 4 Department of Obstetrics and Gynaecology, University of Melbourne, Australia; 5 Department of Surgery, University of Melbourne, Australia

## Abstract

**Background:**

Ovarian cancer is characterized by a wide-spread intra-abdominal metastases which represents a major clinical hurdle in the prognosis and management of the disease. A significant proportion of ovarian cancer cells in peritoneal ascites exist as multicellular aggregates or spheroids. We hypothesize that these cellular aggregates or spheroids are invasive with the capacity to survive and implant on the peritoneal surface. This study was designed to elucidate early inherent mechanism(s) of spheroid survival, growth and disaggregation required for peritoneal metastases

**Methods:**

In this study, we determined the growth pattern and adhesive capacity of ovarian cancer cell lines (HEY and OVHS1) grown as spheroids, using the well established liquid overlay technique, and compared them to a normal ovarian cell line (IOSE29) and cancer cells grown as a monolayer. The proteolytic capacity of these spheroids was compared with cells grown as a monolayer using a gelatin zymography assay to analyze secreted MMP-2/9 in conditioned serum-free medium. The disaggregation of cancer cell line spheroids was determined on extracellular matrices (ECM) such as laminin (LM), fibronectin (FN) and collagen (CI) and the expression of α2, α3, αv, α6 and β1 interin was determined by flow cytometric analysis. Neutralizing antibodies against α2, β1 subunits and α2β1 integrin was used to inhibit disaggregation as well as activation of MMPs in spheroids.

**Results:**

We demonstrate that ovarian cancer cell lines grown as spheroids can sustain growth for 10 days while the normal ovarian cell line failed to grow beyond 2 days. Compared to cells grown as a monolayer, cancer cells grown as spheroids demonstrated no change in adhesion for up to 4 days, while IOSE29 cells had a 2–4-fold loss of adhesion within 2 days. Cancer cell spheroids disaggregated on extracellular matrices (ECM) and demonstrated enhanced expression of secreted pro-MMP2 as well as activated MMP2/MMP9 with no such activation of MMP's observed in monolayer cells. Flow cytometric analysis demonstrated enhanced expression of α2 and diminution of α6 integrin subunits in spheroids versus monolayer cells. No change in the expression of α3, αv and β1 subunits was evident. Conversely, except for αv integrin, a 1.5–7.5-fold decrease in α2, α3, α6 and β1 integrin subunit expression was observed in IOSE29 cells within 2 days. Neutralizing antibodies against α2, β1 subunits and α2β1 integrin inhibited disaggregation as well as activation of MMPs in spheroids.

**Conclusion:**

Our results suggest that enhanced expression of α2β1 integrin may influence spheroid disaggregation and proteolysis responsible for the peritoneal dissemination of ovarian carcinoma. This may indicate a new therapeutic target for the suppression of the peritoneal metastasis associated with advanced ovarian carcinomas.

## Background

'It is not the strongest of the species that survive, or the most intelligent, but the one most responsive to change'–Charles Darwin. Cancer cells are very responsive to their microenvironment and have been shown to acquire resistance in response to physical and chemical stress associated with the changed microenvironment [1]. The vast majority (~90%) of ovarian cancer arises from the malignant transformation of the ovarian surface epithelium [2,3]. This transformation leads to altered adhesion of transformed cells, which in turn results in the shedding of tumor cells into the peritoneal cavity where they float in the peritoneal fluid or ascites as clumps of aggregated cells or spheroids until they find a secondary attachment site for further growth. Even though the attachment of shed floating spheroids to the peritoneal lining and associated organs is the major route for the dissemination of ovarian carcinoma [4], research in ovarian cancer has focused mainly on the metastatic behavior of single cells and little is known about the mechanisms that regulate the survival and peritoneal metastases of shed cancer cells.

Spheroids can be created by culturing different cell lines under conditions where their attachment to matrices is hampered [5]. Such cellular manipulation has been used mostly to understand the mechanism of drug resistance that occurs with *in vivo *three-dimensional growth conditions [6]. As a peritoneal model of metastasis, ovarian carcinoma spheroids have been shown to be protected from apoptosis induced by radiation and common therapeutic drugs such as Taxol [7]. This occurs due to the heterogenous nature of cells within the spheroids, some of which undergo phosphorylation of the anti-apoptotic protein Bad under anchorage-independent settings. Recent studies have demonstrated the capacity of ovarian ascites spheroids to dissaggregate on the mesothelial cells [8-10], yet the mechanism of growth and *in vitro *phenotype of spheroids remain uninvestigated.

The development of peritoneal metastases in ovarian carcinoma is regulated to a large extent by the adherence of shed ovarian tumor cells, as spheroids, to the mesothelial lining of the peritoneum, disaggregation of these cells from the spheroid core and invasion into the extracellular matrix (ECM) of the mesothelial layer. Both cell-cell adhesion and cell-ECM interacting molecules play a role in this process and a number of cell adhesion molecules have been suggested [11]. The foremost important characteristic of a multicellular tumor spheroid is to create an *in vivo *tumor microenvironment that would allow cell-cell association. Hence, the formation of a spheroid depends largely on the expression of certain cell-cell interacting molecules which appear dramatically different in spheroids compared to cells growing as a monolayer [12]. Although the expression of integrins has been studied in various cultured cell lines growing as a monolayer, little is known about the pattern of their expression in cells growing as spheroids. It has been suggested that the inhibition of β1 integrin function can suppress tumor spheroid-ECM interaction in ovarian cancer cell lines [8,13]. A variety of other adhesion molecules have also been implicated in the cell-ECM interaction. The interaction of CD44 to its major ligand hyaluronic acid has been shown to regulate the adherence of ovarian carcinomas to the mesothelium *in vitro *[14] and *in vivo *[15]. CD44 demonstrates a similar adhesive role in gastric and colon cancers [16,17]. Intracellular adhesion molecules (ICAM) have been shown to modulate the adhesion of colon cancer cells to liver endothelial cells [17] and a hepatoma cell line to mesothelial cells [18]. ICAM has also been shown to modulate the adhesion of a hepatoma cancer cell line to the peritoneum [19]. Additionally, a role for the L1-adhesion molecule in peritoneal growth and dissemination of ovarian carcinoma has been recently reported [20]. These studies suggest that integrins and other adhesion molecules play an important role in peritoneal dissemination of cancer cells and understanding such phenomena will help in modulating spheroid growth for better therapeutic outcomes.

An important component of tumor cell invasion involves the enzymatic degradation of the ECM, allowing cancer cells to penetrate the basement membrane and gain access to vasculature to support secondary growth. A complex mixture of proteolytic enzymes mediates this process, including matrix metalloproteinases (MMPs) and serine proteinases (such as urokinase plasminogen activator). MMPs are zinc dependent proteinases and are capable of degrading various ECM components such as collagen, proteoglycans, gelatin, fibronectin, etc. MMPs promote cancer progression by enhancing tumor cell growth, migration, invasion, metastasis and angiogenesis [21]. The roles of MMP2 and MMP9 in the regulation of tumor invasiveness and growth are well established in *in vivo *and *in vitro *animal models [22]. Ovarian cancer cells express MMP2 and MMP9, and we and others have shown that increased expression of MMPs is associated with cancer cell invasiveness and metastatic potential [23]. MMP2 and 9 are expressed in the ascites and plasma of ovarian cancer patients [24]. Experimental studies have shown that animals bearing ovarian carcinoma xenografts in the peritoneal cavity and treated with MMP inhibitors formed less ascites and survived longer [21]. Moreover, stromal MMP9 contributes to the malignant behavior of cancer cells by promoting new vessel sprouting and tumor growth through enhanced expression of VEGF [22]. These studies suggest that MMP2 and MMP9 play an integral role in the progression of ovarian cancer.

In the present study, we have characterized the early growth characteristics, adhesive, invasive and disaggregation capacity of HEY and OVHS1 ovarian cancer cell lines grown as spheroids by a liquid-overlay technique. The growth characteristics of a normal ovarian cell line (IOSE29) grown in a similar fashion were also characterized. In order to determine if intrinsic differences existed in the integrin profile, expressions of different integrin subunits were compared in IOSE29, HEY and OVHS1 cell lines grown as monolayers or as multicellular spheroids. Antibody blockade was used to determine whether such differences facilitate their disaggregation and invasive capacity. Our results demonstrate that an important difference in integrin and MMP2/9 expression exists between ovarian cancer cells grown as monolayers versus those grown as spheroids and that function blocking monoclonal antibodies against α2 and β1 integrin subunits and α2β1 integrin can block the disaggregation and MMP2/9 activation of ovarian carcinoma spheroids. Our findings raise the possibility that α2β1 integrin may represent a valuable therapeutic target in the suppression of intra-peritoneal spread associated with the progression of ovarian cancer.

## Methods

### Cell lines and Media

Two established ovarian cancer cell lines HEY and OVHS1 [25] were used in the study. The human ovarian surface epithelial cell line (IOSE29) [26] transfected with SV-40 antigen was obtained from Dr Nelly Auersperg, University of British Columbia, Canada. This cell line is not immortal and can be maintained in culture for 17–20 passages. ISOE29 cell line is not tumorigenic in mouse and mimics normal ovarian cells in culture [26]. HEY and OVHS1 cell lines were maintained and propagated in RPMI (St Louis, MO, USA), while IOSE29 was cultured in Medium 199/MCDB105 (1:1) (Sigma, St Louis, MO, USA). Both medium were supplemented with 10% fetal bovine serum and 2 mM glutamine (Sigma, St Louis, MO, USA). IOSE29 Cells were incubated at 37°C in 5% CO_2 _and routinely checked for contamination. Viability was checked routinely by the Trypan blue exclusion method.

### Antibodies and reagents

Monoclonal antibodies against integrin subunits α2 (clone P1E6), α3 (clone ASC-6), α6 (clone 4F10), αv (clone AV1) and β1(clone P5D2) were obtained from Chemicon International (Temecula, CA, USA). Monoclonal antibodies against cyclin D2, caspase-3 (4-1-18), phospho-Akt and Akt were from Cell Signalling Technology (Beverly, MA, USA). Antibody against Ki67 was from DAKO Cytomation (Denmark). Phycoerythrin-conjugated goat anti-mouse IgG was obtained from Chemicon International (Temecula, CA, USA). Horseradish peroxidase-conjugated goat anti-mouse antibody was obtained from Bio-Rad (Hercules, CA, USA) while HRP-conjugated donkey anti-rabbit antibody was from Amersham Biotechnology (UK). Fibronectin (FN), laminin (LM) and collagen I (CI) were obtained from Sigma (St Louis, MO, USA).

### Human samples

The study was approved by the Research and Human Ethics Committee (HEC#02/30, 02/29) of the Royal Women's Hospital, Melbourne, Australia. Resected tissues not required for clinical analyses were obtained from patients who presented for surgery at the Royal Women's Hospital, Melbourne, after the provision of a participant information statement and only with informed consent. Normal ovaries, needed for control comparisons were removed from patients undergoing surgery as a result of suspicious ultrasound images, palpable abdominal masses and family history. Histological grading of ovarian carcinoma was performed by two trained staff pathologists using the method described by Silverberg [27].

### Spheroid Culture

Spheroids were created using the liquid overlay technique described previously [1]. Briefly, culture dishes or plates were coated with 0.5%w/v agarose in serum free medium (1:1) and allowed to dry for 30 min. Cells were released from monolayer culture using 0.25% trypsin/0.2% EDTA (JRH Biosciences™, Victoria, Australia), re-suspended in normal medium and layered on agarose. The cultures were maintained at 37°C, 5%CO_2 _for 6 h to 10 days. Additional medium was added at day 7 to maintain nutrient levels.

### MTT assay

Spheroids were created in 12 well plates via the liquid overlay technique described above. Six replica 12-well plates were seeded with 5 × 10^4 ^cells, which were incubated at 37°C, 5% CO_2 _until required for collection at days 1, 2, 3, 4, 7, 8 or 10. On the day of collection, MTT reagent (0.5 mg/ml) was added for 1.5 h. In monolayer cultures, medium was removed from wells and 150 μl solubilizing buffer (1%w/v SDS, 90%v/v dimethyl sulphoxide) was used to dissolve the formazan crystals. For spheroid cultures the formazan crystals were collected by centrifugation (13,000 rpm, 10 min), dissolved in solubilizing buffer and then transferred to 96 well microtitre plates. Samples were read at OD_595 nm _on a Microplate reader (BioRad Model 3550).

### Immunohistochemistry

Spheroids grown in cell culture were harvested by centrifugation then frozen in embedding medium (OCT) using isopentane cooled in liquid nitrogen. Patient tissue was similarly embedded in OCT and frozen in liquid nitrogen cooled isopentane. Blocks were stored at -80°C. Sections were cut at a 5 μm thickness and, if not required immediately, slides were stored at -20°C. For staining, sections were fixed using cold acetone (4°C) for 15 min, transferred to Tris-buffered saline (TBS) pH 7.6, then incubated for 1 h with primary antibody diluted in 1% w/v BSA/TBS. Antibody binding was amplified using CHEMICON IHC Select™ Immunoperoxidase Secondary Detection System according to the manufacturer's instructions and visualized using diaminobenzidine (DAB). Nuclei were counterstained with Mayer's haematoxylin and an IgG1 isotype was used as the negative control.

Sections were assessed microscopically for positive DAB staining. Two observers (KS and CR) independently evaluated the immunostaining results. The concordance ratio was > 95%. Differences of opinion were resolved by reaching a consensus with the assistance of a third evaluator (NA). Four sections were assessed per tissue and tissue and cellular distribution of staining was determined. Parallel frozen sections were stained with hematoxylin and eosin to confirm results.

### Western blotting

Monolayer and spheroid cultures prepared as described above were harvested, washed twice in PBS then snap frozen in liquid N_2 _and stored at -80°C. Prior to sonication, cells were re-suspended in a cell lysis buffer (10 mM Tris, 150 mM NaCl, 2 mM ethyleneglycol tetraacetic acid (EGTA), 2 mM dithiolthreitol (DTT), 1 mM sodium orthovanadate) supplemented with 1 μl/ml aprotinin and 10 μg/ml phenylmethylsulfonyl fluoride (PMSF). Cell debris was the removed by centrifugation (13,000 rpm, 5 min) and supernatant stored at -20°C. Cell lysates containing equal amounts of protein were resolved on 10% or 15% SDS-PAGE gels under non-reducing conditions and transferred to nitrocellulose membranes. Membranes were probed with primary antibody followed by peroxidase-labelled secondary antibody and visualised by enhanced chemiluminescence (ECL) (Amersham, Buckinghamshire, UK) detection system according to the manufacturer's instructions.

### Adhesion assay

Adhesion assays were performed as described previously [28]. Both normal and cancer cell lines grown as monolayers were collected after trypsinization in medium containing 1% serum. Spheroids were trypsinized, harvested by centrifugation and re-suspended in the same medium as the monolayer cells as a single cell suspension. Briefly, 1 × 10^4 ^cells were plated in triplicate on 96 well plates coated with poly L lysine, FN, LM or CI (10 μg/ml) at 37°C for 90 min. Cells were then washed three times with PBS to remove non-adhering cells and the adherent cells were fixed with 100% methanol for 5 min at room temperature. Cells were stained with 0.5% crystal violet for 15 min. Stained cells were washed with PBS, dried and absorbance measured at 595 nm with V_max _plate reader (Bio-Rad, Hercules, CA, USA).

### Spheroid Adhesion and Migration Assay (Disaggregation)

Spheroids were grown in 92 cm dishes as described above. The disaggregation assay was performed as described previously [8]. Briefly, 96-well plates coated with 10 μg/ml FN, LM, CI or bovine serum albumin (BSA) were blocked with BSA (1 mg/ml) for 2 h. Plates were washed with PBS and spheroids suspended in serum-free RPMI medium were layered on the wells at 5–10 spheroids per well. Spheroids were sized and photographed at 1 h, 8 h and 24 h. The fold change in area was calculated by dividing the pixel area of the spheroid at 8 and 24 h by the pixel area at time 0.

### Gelatin Zymography

Gelatin zymography was performed as described previously [29]. Briefly, serum-free medium, concentrated using 10 kDa Amicon Ultra-4 spin columns and containing equal protein loads, was resolved on 10% Tris.HCl acrylamide gels containing 0.1%w/v gelatin. The gel was washed 5 times in zymogram wash buffer [Tris.HCl (pH 7.6), 5 mM CaCl_2_, 1 mM ZnCl_2_, 0.01% N_3_, 2.5% Triton X-100] followed by 3 washes in incubation buffer [Tris.HCl (ph 7.6), 5 mM CaCl_2_, 1 mM ZnCl_2_, 0.01% N_3_] then incubated for 48 h at 37°C in incubation buffer before being stained with coomassie blue (G-250) for visualization of activation. After destain (30% methanol, 1% formic acid), areas void of blue stain indicated areas of enzyme activity. Molecular markers were used to identify pro-MMP2/9 and MMP2/9.

### Immunofluorescence

Cryostat sections were fixed in 4% paraformaldehyde, permeablized in 0.1% Triton X-100, and blocked in 1% BSA. Sections were probed with primary antibody (dilution 1/100 to 1/500) for 2 h followed by 1 h with Alexa Fluor 488 labeled secondary. Sections were counter stained with ethidium bromide (1/10,000) and coverslips were mounted using Fluorgaurd^© ^(BioRad Laboratories, USA) and sealed with nail polish. Fluorescence was imaged using a Leica TCS SP2 AOBS laser confocal microcope (Leica, NSW, Australia) and associated software.

### Flow cytometric analyses

Flow cytometric method was used as described previously [23]. Briefly, monolayer and spheroid cultures of ovarian cancer cell lines were collected and washed twice with PBS. Spheroids were disintegrated into a single cell suspension by 2–3 mins of trypsinization and repeated pipetting. 10^6 ^cells were incubated with primary antibody for 1 h at 4°C and excess unbound antibody was removed by washing twice with PBS. Cells were stained with secondary antibody conjugated with phycoerythrin for 20 min at 4°C, washed twice with PBS and then re-suspended in 0.5 ml phosphate buffered saline (PBS) prior to FACScan analysis. In each assay background staining was detected using an antibody-specific IgG isotype. All data were analysed using Cell Quest software (Becton-Dickinson, Bedford, MA, USA). Results are expressed as mean intensity of fluorescence (MIF).

### Statistical analysis

Student's t-test was used for statistical analyses of proliferation, adhesion, migration and invasion assays. Statistical significance was indicated by p < 0.05. Data are presented as mean ± SEM. Each experiment was repeated three times with a minimum of three replicates.

## Results

### Spheroid formation and growth

**Figure 1:**
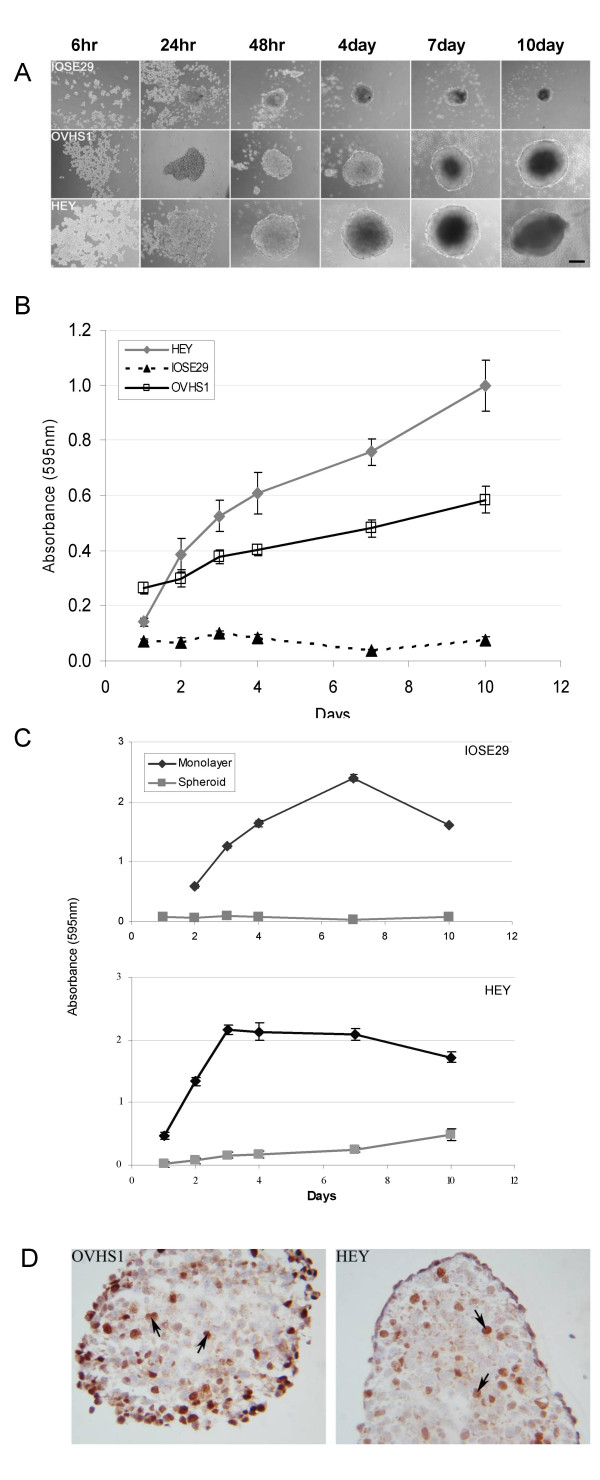
**(A) **Formation of normal and ovarian carcinoma cell line spheroids over 10 days. HEY, OVHS1 and IOSE29 cell lines at a density of 10^4^cells/ml were seeded on 0.5% agarose-coated wells in the presence of normal growth medium for 10 days. Aggregation of cells was viewed using an inverted microscope with phase contrast, magnification 100 ×. **(B) **Proliferation of ovarian carcinoma cells grown as spheroids. HEY, OVHS1 and IOSE29 cells were seeded on agarose-coated plates as described in the Materials and Methods. The level of proliferation was measured by MTT assay as described in the Materials and Methods. Data are representative of three experiments expressed as mean ± SEM of twelve replicates. **(C) **Comparison of proliferation of IOSE29 (upper panel) and HEY (lower panel) cells grown as monolayer versus spheroids. Proliferation was measured by MTT assay as described above and data are expressed as mean ± SEM of six replicates. **(D) **Immunohistochemical staining of Ki67 in day 4 HEY and OVHS1 spheroids. Day 4 HEY and OVHS1 spheroids were collected, embedded in OCT, sectioned and stained as described in the Materials and Methods. Magnification 400 ×.

Ovarian cancer cell lines HEY and OVHS1 and immortalized ovarian surface epithelial (IOSE) cell line IOSE29 were analysed for formation of spheroids and subsequent proliferation when maintained in a suspension culture. Figure 1A illustrates spheroid formation and growth over time and demonstrates that all three cell lines are able to form spheroids. Within 24 h of seeding onto agarose-coated plates, both normal and cancer cell lines clustered and formed cellular aggregates of approximately 400–800 μm for the cancer cell lines and much smaller for the normal ovarian cell line. With time in culture, the cancer cells became tightly packed, rounded and gradually increased in size while the normal ovarian cell line IOSE29 gradually disintegrated, decreasing in size. Detailed morphological analysis of OVHS1 and HEY spheroids using light microscopy revealed that the spheroids of both cell types formed well rounded, compact spheroids with defined margins. On the other hand, cells within IOSE29 spheroids dispersed and by day 10 were drastically reduced in size indicating a characteristic of apoptosis.

Cellular growth of OVHS1, HEY and IOSE29 cell lines was analysed using an MTT assay. In both cancer and normal ovarian cell lines the growth of spheroids was significantly reduced when compared to growth in traditional monolayer culture (Figure 1C). On comparison of spheroid growth only, HEY and OVHS1 spheroids showed a steady increase in metabolic activity (Figure 1B, solid black and grey lines respectively), while IOSE29 maintained only a low level of activity (Figure 1B, dotted line). These data indicate that while the normal ovarian cell line cannot proliferate in suspension culture both ovarian cancer cell lines were able to do so which is consistent with the growth observed morphologically.

The specific ability of cells within the spheroids to contribute to growth was subsequently confirmed using immunohistochemical staining of Ki67 on OVHS1 and HEY spheroid sections. Nuclear staining of Ki67, a standard histological marker for proliferation, identified a population of proliferating cells in spheroids of both OVHS1 and HEY cell lines (Figure 1D). These data suggest that ovarian tumour spheroids are capable of maintaining a proliferating population in suspension cultures.

### Analysis of cell cycle mediators and pro/anti-apoptotic markers in spheroids

**Figure 2:**
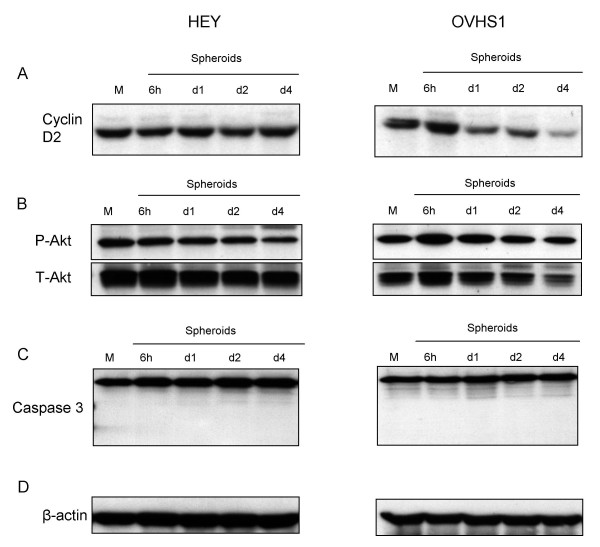
Expression of mediators for cell cycle progression, survival and apoptosis in HEY and OVHS1 cell lysates obtained from cells growing as monolayer or spheroids over 4 days. Cell lysates were prepared and the expression of **(A) **cyclin D2, **(B) **P-Akt, T-Akt and **(C) **caspase-3 was determined by Western blotting as described in the Materials and Methods. Membranes from P-Akt immunostaining were stripped and re-probed for the expression of total Akt. **(D) **Total protein loading was determined by probing the membranes for β-actin. The experiment is representative of three different experiments with similar results.

The ability of spheroid cultures to metabolize and proliferate in culture was correlated by western blot analysis of key mediators for cell cycle progression and pro/anti-apoptotic proteins in spheroids collected at 0 (monolayer control) and 6 h and 1, 2 and 4 days. The D-cyclins are integral in early G1 to S phase transition in the cell cycle [30]. The activation of protein kinase Akt/PKB and caspase-3 play a central role in cell survival and apoptosis, respectively [31-33]. The expression of cyclin D2 was sustained in HEY spheroids but gradually decreased in OVHS1 spheroids (Figure 2A). This is consistent with the MTT response, which was lower in OVHS1 spheroids, compared to HEY spheroids. The activation of Akt was maintained throughout the 4 days of spheroid growth in both HEY and OVHS1 cultures (Figure 2B) while no activation of caspase-3 (Figure 2C) was observed in spheroids of either cell line. These results suggest that both HEY and OVHS1 spheroids are able to maintain their survival response without the induction of ankiosis-dependent apoptosis. Equal protein loading was confirmed for all Western blots using β-actin staining (Figure 2D).

### Spheroid Adhesion

**Figure 3:**
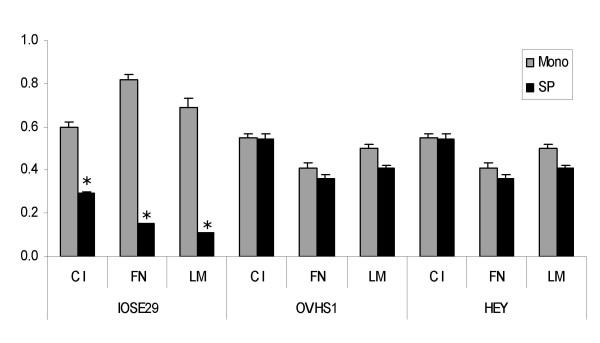
Adhesion of IOSE29, HEY and OVHS1 cell lines grown as monolayer versus spheroids on different ECM. The adhesive response of cells grown as monolayer or as spheroids (day 2) on CI, FN and LM was determined as described in the Materials and Methods. Experiments on HEY and OVHS1 cells were repeated at least three times and performed in triplicate. The experiment on IOSE29 cell line was repeated twice. Results are representative of one experiment performed in triplicate, *significantly different from cells growing as a monolayer, p < 0.05.

In order for spheroids to contribute to cancer metastasis, they must be able to adhere to the extracellular matrix (ECM) of the peritoneal cavity. Compared to monolayer cells, the adhesion of day 2 IOSE29 spheroids decreased by approximately 50% or more (p < 0.05), while adhesion of OVHS1 and HEY remained unchanged with no significant differences on any of the ECM components (Figure 3). Similar results were observed for day 4 spheroids of OVHS1 and HEY (data not shown) indicating sustained adhesive capabilities despite being in suspension cultures for up to 4 days.

### Metastatic Potential

Peritoneal dissemination of ovarian cancer spheroids occurs when cells within the free-floating spheroids attach to the mesothelial lining of the peritoneum and disaggregate, spreading to the secondary site. This process also involves invasion, which requires proteolysis of ECM proteins underlying the mesothelial layer. To understand if cells within the spheroids exhibit such metastatic properties, spheroids were analyzed by *in vitro *disaggregation assay on various ECM components. Similarly, a comparison of proteolytic activity of spheroids versus monolayer cells was examined by testing the expression and activation of MMP2/9 using gelatin zymography.

**Figure 4:**
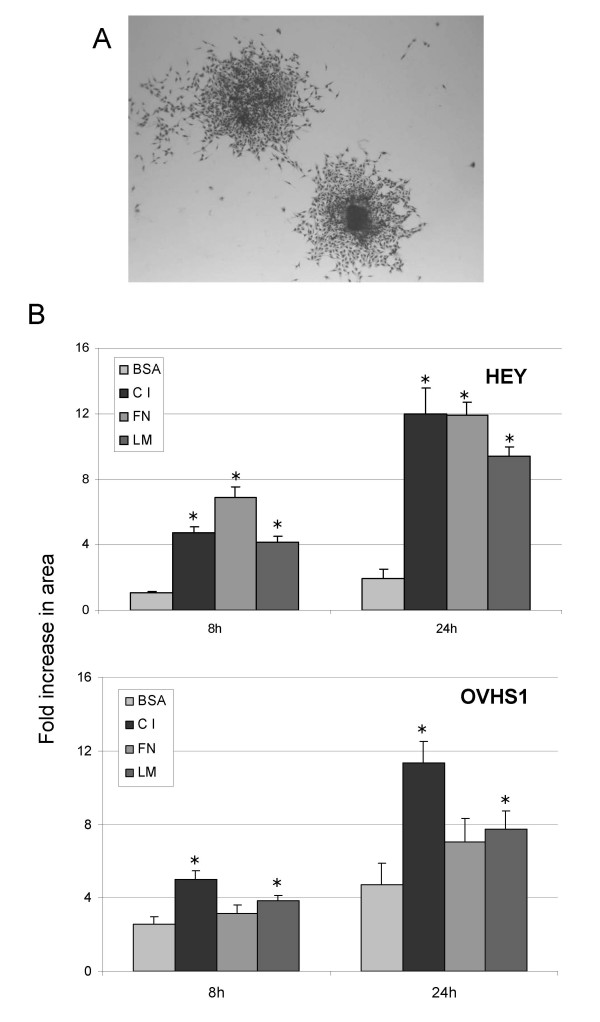
Disaggregation of HEY and OVHS1 spheroids on ECM. HEY and OVHS1 cells were grown on 0.5% agarose for 4 days. Spheroids were collected and allowed to disaggregate on different ECM. **(A) **Morphological feature of disaggregation of HEY spheroids on CI after 24 h. **(B, C) **HEY and OVHS1 cells grown as spheroids for 4 days were allowed to adhere and then disaggregated on BSA, CI, FN and LM and photographed at 1 (control), 8 and 24 h. The extent of disggregation was measured as described in Materials and Methods. Values shown represent the average fold change in pixel area of > 10 spheroids over 8 and 24 h from two experiments, ± SEM, *p < 0.05, significantly different from disaggregation on BSA.

OVHS1 and HEY spheroids began to transform from a three-dimensional structure into flattened cell clusters within 8 h. Figure 4A illustrates two disaggregating spheroids after 24 h on a collagen coated plate. As the cells migrated away from the core of the spheroid, cell-cell contact was reduced and adherence and spreading on the ECM constituents occurred. Within 24 h of cells attaching to the ECM matrices (FN, LM and CI), both OVHS1 and HEY spheroids (day 4) exhibited an increase in spheroid disaggregation when compared to a BSA control. In HEY spheroids, a significant increase in disaggregation was observed as early as 8 h on all matrices tested, while for OVHS1, only CI and LM facilitated significant disaggregation (p < 0.05).

**Figure 5:**
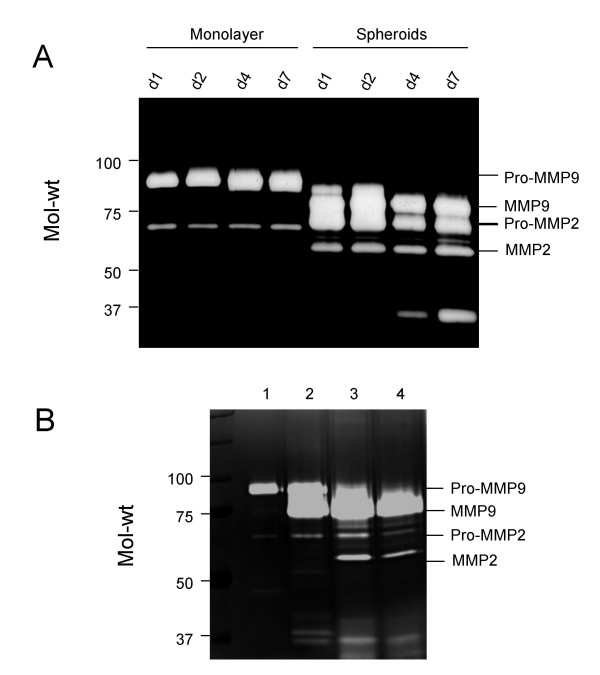
**(A) **Expression of secreted pro-MMP2/9 and MMP2/9 in the cell free medium of HEY cell line grown as a monolayer or as spheroids over 7 days. Serum-free medium from the cells were collected and concentrated as described in the Materials and Methods. 1 μg of protein from cells grown as a monolayer and 0.5 μg of protein from spheroids was resolved on a 10% polyacrylamide gel supplemented with 1% gelatin. The experiment was repeated on OVHS1 cells with similar results. **(B) **Expression of secreted pro-MMP2/9 and MMP2/9 activity in the cell free medium of HEY cell line grown as a monolayer in the absence or presence of plasminogen (10 ng/ml, lanes 1 and 2 respectively) and as spheroids over 4 days in the absence or presence of 1:10 phenanthroline (2 mM, lanes 3 and 4 respectively).

Serum-free medium (SFM) was collected from monolayer cells and cells grown as spheroids from day 1 to 7. In monolayer cultures, only the expression of pro-MMP2/9 was observed whereas in spheroids the expression of active MMP2 and MMP9 was induced and pro-MMP9 expression was replaced with active MMP9 (Figure 5A). The identity of active MMP2 and MMP9 was determined by treating SFM from a HEY monolayer culture with plasminogen (10 ng/ml) overnight. Plasminogen activated pro-MMP9 (Figure 5B, lane 1) to active MMP9 (Figure 5B, lane 2). On the other hand, inhibition of active MMP2 (Figure 5B, lane 3) was observed when SFM from day 2 HEY spheroid culture was treated with 1:10 phenanthroline (2 mM) overnight (Figure 5B, lane 4) [34]. In the presence of phenanthroline no significant inhibition of active MMP9 was observed. The identity of the 37 kDa band in day 4 and 7 spheroids (Figures 5A and 5B) is not known but has been observed by others in ovarian cancer cells [35].

### Cell surface expression of α-integrin subunits

**Figure 6:**
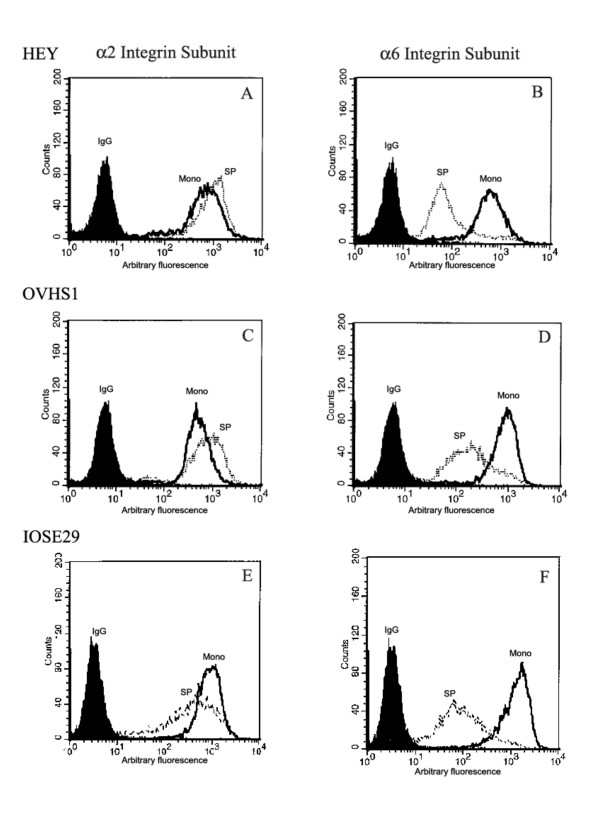
Flow cytometric analyses of α2 and α6 integrin subunits in HEY **(A and B)**, OVHS1 **(C and D) **and IOSE29 **(E and F) **cells grown as a monolayer and as spheroids (4 days for HEY and OVHS1 cells and 2 days for IOSE29 cells). Cells were incubated with either control IgG or primary α2 or α6 monoclonal antibody followed by secondary goat anti-mouse IgG conjugated with phycoerythrin. The mean intensity of fluorescence (MIF, arbitary units, log scale) was measured. The filled histogram in each figure is control IgG, black lines indicate the expression of protein in monolayer cells while broken lines demonstrate protein expression within the cells in spheroids. Results are representative of three independent experiments. Monolayer = mono, spheroid = Sp

Cell migration and invasion is facilitated by the cell-surface expression of specific integrins. Expression of α2, α3, α6, αv, and β1 integrin subunits was determined in spheroids over 4 days of culture and compared to that of monolayers. The expression of α3, αv and β1 integrin subunits remained unchanged, however, a significant increase was observed in the expression of α2 integrin within 24 h of spheroid culture and there was a decrease in the expression of α6 integrin subunit expression over the 4 days of culture (Table 1, Figure 6). Both HEY and OVHS1 cells do not express β4 integrin subunit. The expression of α2, α3, α6 and β1 integrin subunits was decreased in IOSE29 cells by 1.5–7.5-fold within 2 days while the expression of αv integrin subunit was sustained (Figure 6 and Table 2). This is consistent with the significant loss of adhesion in cells forming IOSE29 spheroids seen within 2 days as described above. These data suggest that there is a difference in the modulation of integrin expression in spheroids of cancer versus normal cells and that this explicit difference may form the basis of longer survival of cancer cells in anchorage independent conditions compared to normal cells.

**Table 1:** Expression of integrin subunits in OVHS1 and HEY cells growing as a monolayer and as spheroids for 4 days

Cell Type	OVHS1		HEY	
Integrin subunit	Monolayer (MIF)	Spheroid day 4 (MIF)	Monolayer (MIF)	Spheroid day 4 (MIF)

α2	509 ± 47	735 ± 41*	625 ± 40	886 ± 22*
α3	932 ± 60	905 ± 99	1107 ± 108	912 ± 112
αv	96 ± 1	114 ± 5	125 ± 14	120 ± 10
α6	661 ± 93	151 ± 18*	418 ± 45	208 ± 20*
β1	1302 ± 205	1340 ± 44	1224 ± 123	1072 ± 8

Values are expressed as mean intensity of fluorescence (MIF)

± SEM of three different readings obtained from independent experiments.

*Significantly different from cells growing as a monolayer (p < 0.05).

**Table 2:** Expression of integrin subunits in IOSE 29 cells growing as a monolayer and as spheroids for 2 days

Integrin subunit	Monolayer (MIF)	Spheroid day 2 (MIF)
α2	830 ± 91	542 ± 142
α3	889 ± 69	335 ± 34*
αv	105 ± 84	95 ± 14
α6	1332 ± 171	176 ± 64*
β1	701 ± 84	294 ± 54*

Values are expressed as mean intensity of fluorescence (MIF)

± SEM of three different readings obtained from independent experiments.

*Significantly different from cells growing as a monolayer (p < 0.05).

**Figure 7:**
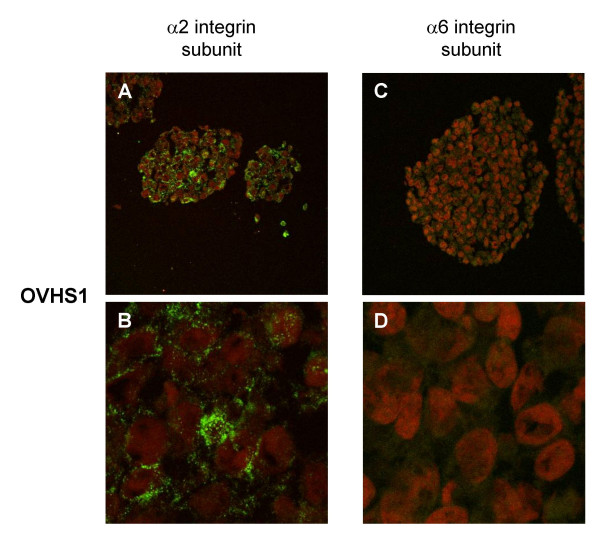
Cellular localisation of α2 and α6 integrin subunits in spheroids grown for 4 days. Using Alexa-fluor immunofluorescence, spheroids embedded in OCT were sectioned and stained for α2 and α6 integrin subunits and counterstained as described in the Materials and Methods. Images captured at magnification 400 × using an oil immersion lens on a Leica con-focal microscope. B and D are 1.5 × magnification of A and C respectively

Cellular expression of α2 and α6 integrin subunits was further investigated by confocal microscopy on spheroids of OVHS1 cell line (Figure 7). Stronger expression of α2 integrin subunit was observed in the peripheral cells lining the outer layer of the spheroids (Figure 7A). On higher magnification, expression of α2 integrin subunit was evident on the cell-cell interface (Figure 7B). In contrast, the expression of α6 integrin was relatively low and diffuses in localization (Figures 7C and 7D).

### α2β1 integrin and spheroid proteolysis/disaggregation

**Figure 8:**
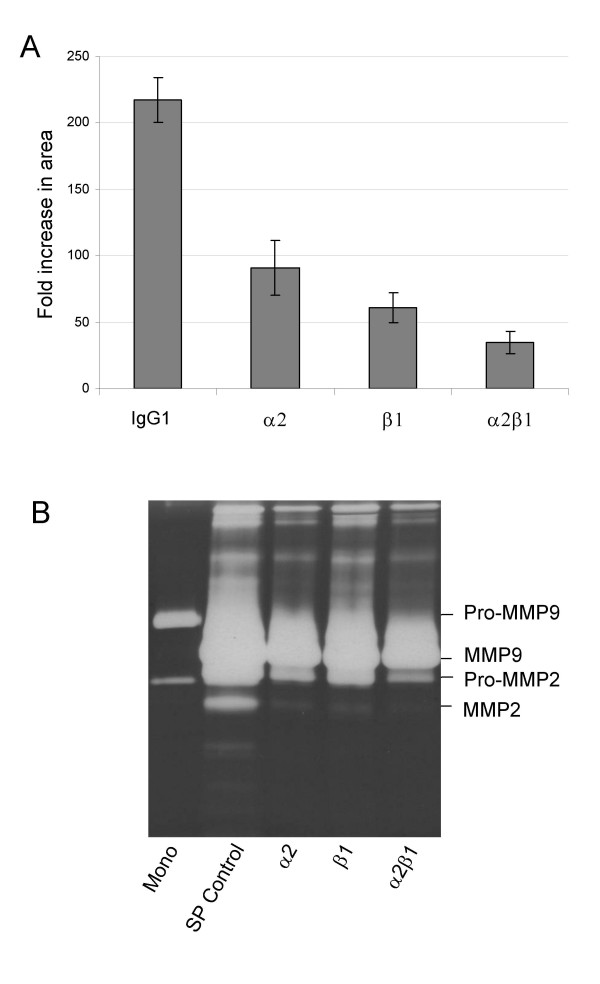
Effect of blocking α2, β1 subunits and α2β1 integrin on **(A) **disaggregation of 4 day spheroids on CI, and **(B) **MMP2/9 activation in HEY spheroids grown on 0.5% agarose for 2 days. To determine the neutralizing effect of antibodies spheroids were treated with the antibodies (20 μg/500μl) for 30 min before plating on CI for disaggregating assay. For gelatin zymography cells (10^6^/ml) were treated with the antibodies (20 μg/ml) before seeding on 0.5% agarose in SFM fror 48 h. The experiment was repeated on OVHS1 cells with similar results.

To investigate if α2β1 integrin has any effect on the migration and proteolysis of spheroids, disaggregation and gelatin zymography analysis were performed in the presence of blocking antibodies. Since α2β1 is a collagen receptor, spheroid disaggregation was performed on CI- coated plates using blocking antibodies against α2, β1 and α2β1. Disaggregation of both OVHS1 and HEY spheroids (day 4) was significantly reduced by α2, β1 and α2β1 blocking antibodies (p < 0.05) with the greatest inhibition seen in the presence of anti-α2β1 integrin (Figure 8A). Under the same blocking conditions the activation of MMP-2 was also reduced (Figure 8B). The effect of inhibitory antibodies on the activation of MMP9 under similar conditions was difficult to discern because of the relatively high concentration of MMP9 in the medium. Taken together these data indicate that α2β1 integrin may have a role in maintaining the migration and invasive potential of spheroids.

### Expression of α2 and α6 integrin subunits in normal ovaries, high-grade ovarian tumors and in patient's ascites

**Figure 9:**
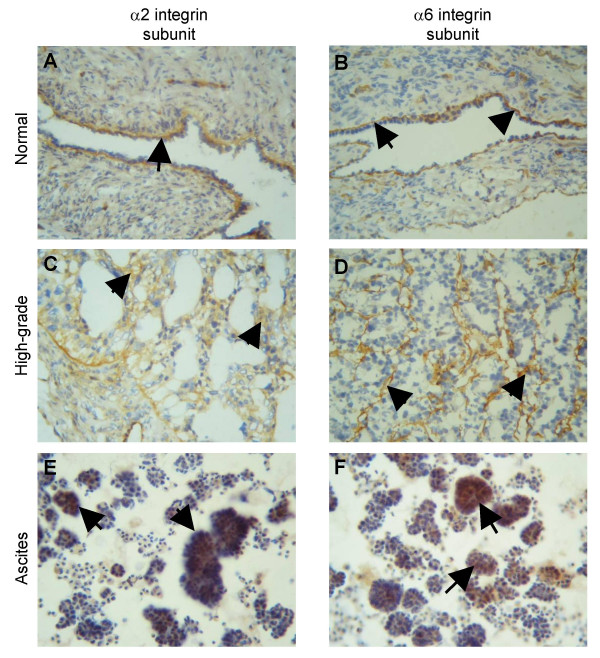
Expression of α2 and α6 integrin subunits in **(A and B) **normal ovaries, **(C and D) **high-grade ovarian tumors and **(E and F) **cellular aggregates present in a patient ascites. Cryostat sections of ovarian tissues and smears of ascites were stained by the immunoperoxidase method for the expression of α2 and α6 subunits as discussed in the Materials and Methods. **(A and B) **Normal ovaries, arrow showing continuous epithelial expression of α2 and α6 integrin subunits, **(C and D) **grade 3 serous ovarian tumors, arrows indicating irregular expression of α2 and α6 integrin subunits in epithelial cells; **(E and F) **arrows indicate cluster of epithelial tumor cells in a patient's ascites staining for α2 and α6 integrin subunits. Magnification 400 ×.

In the normal ovarian tissues (n = 10) the expression of α2 and α6 integrin subunits was confined to the basal layer of epithelial cells and displayed continuous labeling (Figures 9A and 9B). Staining of endothelial cells lining the blood vessels was also observed and in a few cases stromal staining was also evident. Conversely, scattered heterogeneous epithelial staining of α2 and α6 integrin was observed in high-grade ovarian tumors (grade 3, n = 13, 10 serous, 1 endometrioid and 2 clear cell carcinoma subtype) (Figures 9C and 9D). In all malignant tumors, the basal reactivity of the epithelial layer was present in a discontinuous fashion. Stromal staining as well as staining of endothelial cells was also evident in some tumor sections.

Immunohistochemical staining of ascites smears (n = 4) from high-grade ovarian cancer patients demonstrated strong staining for α2 integrin subunits in clusters of malignant cells. Some α6 subunit staining was present in malignant cellular aggregates but it was weaker than α2 subunit staining. Weak staining of α2 and α6 subunits in some single cells (epithelial cells, mesothelial cells, inflammatory cells, etc) was also present. These results suggest that α2 and α6 integrin is expressed by aggregates of malignant cells present in ascites.

## Discussion

Some recent studies have demonstrated that a significant proportion of ovarian cancer cells in ascites exist as multicellular aggregates and have the capacity to adhere and invade the mesothelial cells lining the peritoneum [9,10]. Free floating multicellular aggregates or spheroids in the ascites of cancer patients are difficult to isolate as they break during purification and in some instances fail to attach to and proliferate on a tissue culture substratum [36]. Hence, to improve our understanding of spheroid growth and spread with the perspective of developing effective therapeutic targets for advanced-stage ovarian cancer, we have developed an *in vitro *spheroid model which mimics *in vivo *multicellular spheroids in the peritoneal effusions of women with ovarian cancer. The ability of spheroids to contribute to the spread of cancer has been assessed by growth, adherence and disaggregation capabilities and by investigating the profile of integrins and MMP2/MMP9 that may mediate the dissemination process. In some instances comparisons have been made to a normal ovarian cell line grown under similar conditions.

Cancer cell line spheroids resembled those present in the ascites of cancer patients. Spheroids formed from ovarian cancer cell lines increased in size with time, and formed compact regular spheroid structures. As a product of anchorage-independent culture, multicellular spheroids had decreased proliferative abilities compared to the cells cultured as monolayers. Hence, the monolayer cultures approached confluence within seven days and decreased proliferation, while the slower growing spheroids continued to grow for at least 10 days. This response was supported by staining for the proliferation marker Ki67, which demonstrated the presence of proliferating cells throughout both HEY and OVHS1 spheroids. In addition, cancer cell line spheroids sustained activation of Akt, expression of cyclin D2 and did not display any activation of caspase-3. On the other hand, the normal ovarian cell line (IOSE29) failed to proliferate under similar conditions and its growth response was consistent with reduced size and disintegration of spheroids with time. This disparity in the response of normal versus cancer cell lines in response to anchorage-independent surroundings reflects major differences in the cohesive response required for cell-cell contact in order to maintain survival. Expression of Akt is amplified in many cancers, including ovarian cancer [31]. Akt kinase activity is high in ovarian cancer tissues and is associated with undifferentiated histology and aggressive clinical behaviour, suggesting that Akt contributes to tumor progression [37]. Akt kinase promotes cell survival by activating pro-apoptotic Bad, which in its activated state binds anti-apoptotic agents, Bcl2 or Bclxl that either on its own or together binds Bax, inhibiting the release of cytochrome C from the mitochondria and activation of caspase-3 [38]. Therefore, by maintaining Akt kinase activity, ovarian cancer spheroids inhibit caspase-3 activation and, subsequently, apoptosis.

We and others have shown that ovarian cancer cell lines have the ability to adhere to ECM proteins such as FN, LM, CI, etc [39]. Some studies have shown that spheroids generated from the NIH-OVCAR5 cell line can adhere to type IV collagen, FN and LM [40] and adhesion levels of spheroids isolated from patient's ascites were found to be lower than single ovarian cancer cells growing in culture [9]. Our results however, demonstrate no change in the adhesion of cells within the HEY and OVHS1 spheroids when compared to those growing as a monolayer. On the other hand, the normal ovarian cell line (IOSE29) loses its adhesive ability within 2 days, upon acquisition of spheroid morphology. These differences in the adhesion of normal versus cancer cells implicate differences in cell-ECM interaction necessary for establishing a heterotypic interaction with the matrix during the adhesion process.

The peritoneum, omentum and the bowel surfaces are the frequent sites for implantation of metastatic ovarian cancer cells. The outer lining of these metastatic sites is comprised of a single layer of mesothelial cells, which express a variety of ECM proteins, including LM, FN, CI and hylauronan to which tumor cells can adhere before spreading [13,41]. Significant increase in the disaggregation of ovarian cancer spheroids on different matrices reflects the ability of cells within the spheroids to migrate to distant sites. The differences in disaggregation observed in the spheroids obtained from different cell lines, may reflect variability in the expression of cell receptors and is likely to be cell type specific.

In order for the spheroids to disseminate to a secondary site they not only need to adhere, disaggregate and migrate but they also need to invade the mesothelial cell layer to form a stable secondary growth [36]. MMPs play an important role in the invasion of cancer cells and are involved in the degradation of ECM proteins allowing cancer cells to migrate to a secondary site. We report that ovarian cancer spheroids secrete much greater amounts of both pro-MMP2 and MMP9 compared to cells grown as a monolayer, and in the case of spheroids, both MMP2 and MMP9 were present in the active form, while monolayer cells only secreted the inactive precursors. These results are consistent with the abundant amount of MMP2/MMP9 reported in the ascites of ovarian cancer patients [24] and are consistent with the previous study that showed a blockade of spheroid invasion to mesothelial monolayer in the presence of GM 6001, a broad-spectrum matrix metalloproteinase inhibitor [8]. Recently, another study has reported the invasive characteristics of spheroids isolated from the ascites of ovarian cancer patients and has correlated that invasiveness with a shortened survival of ovarian cancer patients by 16 to 17 months [10]. The same study also reported retraction of the mesothelial layer at the site of spheroid attachment. This effect, however, disappeared by day 7, upon complete spheroid cell dispersal, indicating that ascites tumor spheroids may be involved in the degradation of mesothelial monolayer but once disaggregated lose the capacity to do so. While we have not shown invasion of a mesothelial monolayer by spheroids, enhanced secretion of MMPs by HEY and OVHS1 spheroids, clearly implicates the greater invasive capability of spheroids *in vitro*.

The biological mechanism(s) by which spheroids are formed and sustained is not known. Both HEY and OVHS1 cell lines express a variety of integrin receptors. We report an enhanced expression of α2 and a decrease in α6 integrin when comparing spheroidal cells to cells grown as a monolayer, changes which were observable within 24 hours. While the increased expression of α2 was sustained in spheroids for 4 days, α6 expression gradually decreased over the same period of time (data not shown). On the other hand, no change in α3, αv and β1 subunits were observed. In IOSE29 spheroids however, except αv, there was a decrease in the expression of integrin subunits ranging from 1.5–7.5-fold. These results were supported by immunofluorescence studies performed on cancer spheroids that displayed distinct high α2 subunit expression at the periphery of the spheroid and at the outer membraneous layer forming the cell-cell interface of aggregated cells. Very little or no α6 subunit staining was evident in the cell-cell contact regions within the spheroids suggesting very low expression. Based on these observations one can conclude that cellular aggregation and the environmental factors within the spheroids can regulate the expression and localization of a specific sub-set of integrins. Differences in the α2 and α6 integrin expression between monolayer cells and spheroids however, had no effect on their adhesion capabilities on LM or CI suggesting that α2 subunit up regulation may compromise diminution of expression of α6 integrin subtype and hence adhesion on LM. It is reasonable to speculate that in a spheroid scenario it is more cell-cell rather than cell-ECM interaction that will influence integrin expression profile.

To assess if increased α2 expression had any effect on spheroid function, spheroids were treated with blocking antibodies for α2 and β1 subunits and α2β1 integrin and then tested for disaggregation on CI-coated plates. Disaggregation was reduced for all three antibodies, with an apparent accumulative inhibition occurring when α2β1 integrin function was blocked in comparison to individual α2 and β1 subunit function. Parallel to that, blocking α2 and β1 subunits and α2β1 integrin also inhibited activation of MMP2, with no observable change in the expression of pro-MMP's. Although the specific participation of individual integrins in spheroid phenotype is not understood, our results suggest that α2β1 integrin may have a role in the disaggregation and invasion of ovarian carcinoma spheroids.

Selective regulation of integrin receptors in spheroids of squamous cell carcinoma has been reported previously [12]. In that study, the diminution of α6 and β1 integrin subunit levels was observed in spheroids compared to cells grown as a monolayer while no change in the expression of α2, α5 and β5 subunits was shown. Consistent with that, the expression of α6 integrin has been shown to be less in ovarian carcinoma effusions compared to that in the tissues [42]. Recently, interaction between the α5β1 integrin and fibronectin has been shown to mediate the formation of ovarian carcinoma spheroids [40]. In our study we have not compared the expression of α5β1 integrin between monolayer cells and spheroids.

Strong expression of α2 and α6 integrin was observed at the basal layer of surface epithelial cells of normal ovaries. In ovarian tumors there was a loss of regular basement membrane structure resulting in irregular staining of α2 and α6 subunits. Intense staining of α2 integrin was observed in ascites spheroids while staining of α6 subunit occurred to a lesser degree. As α2β1 and α6β1 are the major collagen and laminin receptors on basement membranes, one can speculate that a tumor-induced irregular pattern of matrix-modelling can result in the irregular distribution of these subunits in cancer. In ovarian carcinoma, the expression of α6 subunit has been shown to correlate with the expression of basement membrane protein laminin [42]. The same study also showed decreased or loss of staining of laminin and α6 subunit in malignant cells in ascites suggesting that laminin expression may regulate the expression of α6 integrin subunit [42]. The loss of laminin in the ascites of patients may be due to less synthesis of this basement membrane by tumor cells or may be due to degradation by the proteolytic enzymes secreted by the cells. This deficit in laminin may signal the tumor cells to decrease their α6 subunit expression, consistent with the gradual decrease in α6 subunit expression and high proteolytic activity seen in spheroids.

The spread of ovarian carcinoma is unique as it involves localized invasion and is rarely dependent on dissemination through lymphatics. In this context, the role of shed tumor cells forming spheroids, implantation onto the mesothelial lining of the peritoneum with consequent disaggregation and dissemination is not well understood. As little is known about the ascites tumor cell aggregates or spheroids and the fact that these cells are often dismissed as non-metastatic and undergoing apoptosis is somewhat disturbing. Better outcomes for ovarian cancer patients can only be projected if a targeted approach can be accomplished to disrupt the invasive processes of spheroids requisite for peritoneal spread. Hence, a more comprehensive understanding of ascites spheroid biology is needed to combat the dissemination of ovarian carcinoma. In this study, we aimed to address this issue by characterizing an *in vitro *model for the dissemination of ovarian carcinoma. Using this model we were able to show that α2β1 integrin is up regulated in the spheroids and that functional blockade using monoclonal antibodies reduced the extent of disaggregation and proteolysis of spheroids. These data suggest that molecules that regulate α2β1 integrin functions may have a potential role in inhibiting the invasiveness of peritoneal tumor aggregates or spheroids and may aid in suppressing the dissemination of ovarian carcinoma.
